# Biohydrogen Production in Microbial Electrolysis Cell Operating on Designed Consortium of Denitrifying Bacteria

**DOI:** 10.17113/ftb.61.01.23.7496

**Published:** 2023-03

**Authors:** Putty Ekadewi, Rita Arbianti, Cristina Gomez, Tania Surya Utami

**Affiliations:** 1 Bioprocess Engineering, Department of Chemical Engineering, University of Indonesia, Kampus Baru UI, 16424 Depok, Indonesia; 2 Department of Biotechnology, Indonesia International Institute for Life Sciences, Jl. Pulomas Barat Kav. 88, 13210 East Jakarta, Indonesia; 3 Nantes Université, GEPEA UMR 6144, 37 Bd de l’Université, 44600 Saint-Nazaire, France; 4 Applied Mathematics School, Getulio Vargas Foundation, Praia de Botafogo, 190, 22250-90 Rio de Janeiro, Brazil

**Keywords:** biohydrogen, denitrifying bacteria, microbial community, microbial electrolysis cells, methanogenesis

## Abstract

**Research background:**

This study provides insight into the use of a designed microbial community to produce biohydrogen in simple, single-chamber microbial electrolysis cells (MECs). The ability of MECs to stably produce biohydrogen relies heavily on the setup and microorganisms working inside the system. Despite having the most straightforward configuration and effectively avoiding costly membranes, single-chamber MECs are prone to competing metabolic pathways. We present in this study one possible way of avoiding this problem using characteristically defined, designed microbial consortium. Here, we compare the performance of MECs inoculated with a designed consortium to MECs operating with a naturally occurring soil consortium.

**Experimental approach:**

We adapted a cost-effective and simple single-chamber MEC design. The MEC was gastight, 100 mL in volume, and equipped with continuous monitoring for electrical output using a digital multimeter. Microorganisms were sourced from Indonesian environmental samples, either as denitrifying bacterial isolates grouped as a designed consortium or natural soil microbiome used in its entirety. The designed consortium consisted of five species from the *Pseudomonas* and *Acinetobacter* genera. The headspace gas profile was monitored periodically with a gas chromatograph. At the end of the culture, the composition of the natural soil consortium was characterized by next generation sequencing and the growth of the bacteria on the surface of the anodes by field emission scanning electron microscopy.

**Results and conclusions:**

We found that MEC using a designed consortium presented a better H_2_ production profile, with the ability of the system to maintain headspace H_2_ concentration relatively stable for a long time after reaching stationary growth period. In contrast, MECs inoculated with soil microbiome exhibited a strong decline in headspace H_2_ profile within the same time frame.

**Novelty and scientific contribution:**

This work utilizes a designed, denitrifying bacterial consortium isolated from Indonesian environmental samples that can survive in a nitrate-rich environment. Here we propose using a designed consortium as a biological approach to avoid methanogenesis in MECs, as a simple and environmentally friendly alternative to current chemical/physical methods. Our findings offer an alternative solution to avoid the problem of H_2_ loss in single-chamber MECs along with optimizing biohydrogen production through bioelectrochemical routes.

## INTRODUCTION

Bioelectrochemical systems (BES), more widely known as their derivatives microbial fuel cell (MFC) and microbial electrolysis cell (MEC), are electrochemical cells that utilize microorganisms to carry out reduction/oxidation reactions. Microorganisms responsible for the process can be referred to as electroactive bacteria, exoelectrogens, or anode/cathode-respiring bacteria. These organisms are collectively called electroactive bacteria because of their unique ability to transport electrons through biological membranes either from or to the environment (*1*). BES as a technology platform has been studied only recently, within the past two decades. In this field, the research focus has varied among optimization of the operational conditions of the system, classical study of electroactive microorganisms, or the design of the platform itself. BES has been studied for many applications, including wastewater treatment, fuel gas production as H_2_ and CH_ 4_, nutrient removal and recovery, chemical synthesis, desalination and bioremediation (*2*–*5*).

Despite being coined as the future of clean energy, the majority (around 95%) of produced H_2_ is obtained from fossil fuels through chemical conversion routes (*6*). The majority of H_2_ is produced through thermal processes of natural gas or biomass, *i.e.* steam reforming and gasification. Alternatively, H_2_ can be obtained through water-splitting methods like electrolysis or photolysis of H_2_O (*7*). The research focus of the H_2_ production is now on increasing process efficiency and better economics (*8*, *9*). However, to meet the demand for a cleaner H_2_ production method, bioprocesses have emerged with alternative processes like fermentation and bioelectrolysis (MEC) to generate biohydrogen as end-product with advantages of moderate operational parameters, lower energy requirements and better environmental footprints than fossil resources (*4*, *7*).

When using MEC to produce H_2_, single-chamber configuration was proposed as a solution to avoid the higher cost incurred by the use of membranes found in two- or multi-chamber BES, as well as to reduce resistance due to the presence of a physical barrier between compartments (*10*). In a single-chamber configuration, both anodes and cathodes are located in the same space. Another advantage of the lack of membrane is reduced energy loss and higher energy recovery efficiency (*4*). However, since there are no practical barriers like in the multi-chamber configuration, difficulties can be met in the production of several end-products due to purity issues and product transformation to other unwanted metabolites. For example, in MEC operated under anaerobic conditions, the occurrence of methanogenesis greatly hinders effective biohydrogen production (*10*). Methanogens are responsible for this phenomenon. These microbes are obligate anaerobic microorganisms able to produce CH_4_ out of H_2_ or carbon substrate (*11*). High methanogenic activity is one of the most commonly reported causes of failure for MEC (*11*–*14*), along with the fact that most large MECs use wastewater, which may play a role in their low performance (*15*). As a result, there is an obvious need to improve H_2_ recovery in MECs.

Methanogenesis and denitrification have a very complex relationship. Previous studies have examined their interactions in natural and synthetic environments (*16*, *17*). Overall, methanogenic bacteria were inhibited by the activity of denitrifying bacteria. The inhibitory effect of denitrification on methanogenesis opens the door to exploiting denitrifying bacteria as a control method to suppress the growth of methanogenic bacteria in MEC.

Traditionally, bioelectrochemical cells rely on microbe-rich inocula to fulfil their goals, most notably using digested sludge since complex microbial communities perform better in this setting (*18*, *19*). The use of a designed consortium is a developing research topic in metabolic engineering. In the bioelectrochemical field, designed consortia were used previously to study interspecies electron transfer mechanisms in biogas digestors (*20*) as well as to demonstrate the synergistic effect of two species (*Pseudomonas aeruginosa* PA14 and *Enterobacter aerogenes*) on electricity generation (*21*). Previously, co-culturing *Shewanella oneidensis* with *Escherichia coli* in MFC resulted in higher electrical output with the synergistic effect forming in a short time (*22*). A recent study has shown positive interaction between *Geobacter sulfurreducens* and *Ethanoligenes harbinense* in a co-culture for H_2_ production in a single-chamber MEC, although methanogenesis is not discussed there (*23*). He *et al.* (*24*) also discussed that metabolic engineering approaches like co-culturing bacteria capable of metabolizing CH_4_ along with electroactive bacteria may be the future alternative method of suppressing methanogenesis. This shows that metabolic engineering at the community level is under active research for MECs.

In the past years, our research group has identified nine native microbes from 19 isolates found in local environmental soil and water samples that are spread among two genera: *Acinetobacter* and *Pseudomonas* (*25*). High-throughput screenings of these microbes suggest varying abilities for denitrification and exoelectrogenic activity. This study proposes the use of a designed consortium instead of an uncharacterized microbiome commonly used in bioelectrochemical cells. For this research, we would like to develop functional communities out of our denitrifying isolates to enhance biohydrogen production in MECs by avoiding the transformation of H_2_ in other competing metabolic pathways, notably its transformation to CH_4_ by methanogenesis. We expect that by reducing the complexity of the inoculum, headspace H_2_ concentration can be sustained despite working in a simple single-chamber MEC setup.

## MATERIALS AND METHODS

### Microorganisms and media

Microorganisms used in this study were retrieved from glycerol stock (-80 ºC) from a previous study on microbial isolation and characterization from environmental samples in West Java and Jakarta, Indonesia (*25*). Isolation was carried out on nitrate-rich liquid media under anaerobic conditions prior to colony selection on R2A agar plates (*25*). The isolates were checked for their possession of three central denitrification genes (*nirS*, *nirK* and *nosZ*) using primers available in the literature (*26*) and identified by 16S rRNA sequencing with primer pair 27F/1492R. Isolates were identified by BLASTn (bacteria and archaea) NCBI database (*27*) (https://www.ncbi.nlm.nih.gov/ ), aiming at a lower threshold identity of 98% (*25*). Soil bacteria were sampled from Depok, West Java, Indonesia, without any prior characterization.

Macronutrients were formulated following Bellini *et al.* (*28*), giving the composition of the media at: 1.641 g CH_3_COONa, 0.02 g yeast extract, 0.85 g NaNO_3_, 1.0 g NaCl, 0.5 g NH_4_Cl, 0.0795 g CaCl_2_·2H_2_O, 2.7 g K_2_HPO_4_, 1.3 g KH_2_PO_4_, 0.235 g MgCl_2_·6H_2_O, 40 µL ethanol, 10 mL trace element solution, 10 mL vitamin solution (MEM vitamin solution; Sigma-Aldrich, Merck, St. Louis, MO, USA), 2 mL 0.2% resazurin indicator solution (Sigma-Aldrich, Merck), and distilled water to 1 L. Trace element solution was modified from Bellini *et al.* (*28*) from the initial formulation of Touzel and Albagnac (*29*). The solution consisted of: 1.24 g Titriplex III, 1.35 g FeCl_3_·6H_ 2_O, 0.1 g MnCl_2_·4H_2_O, 0.1 g CaCl_2_·2H_2_O, 0.1 g ZnCl_2_, 0.01 g H_3_BO_3_, 0.024 g Na_2_MoO_4_·2H_2_O, 1.0 g NaCl and distilled water to 1 L. The MECs were filled with the medium prior to autoclaving. Heat-sensitive materials were sterilized by injection into each setup with 0.2-µm filters (hydrophilic Nylon_66_ sterile syringe filter; Axiva Sichem Biotech, Delhi, India) once the reactors cooled down. Medium replenishment at 10% liquid volume of the setup was carried out once in the culture at the first current drop to 0.01 mA.

### Bioelectrochemical system setup

**Fig. 1:**
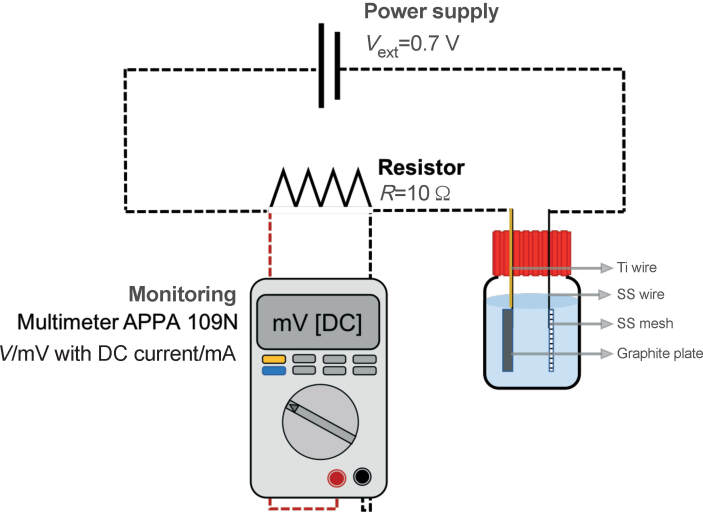
Experimental setup. The reactor is 100 mL in working volume and gastight. Parallel operation was chosen to maintain equal *V*_ext_ to all microbial electrolysis cells (MECs). Multimeter was connected between the anode and the power supply, over a 10 Ω resistance, for continuous monitoring of electrical output. SS=stainless steel; Ti=titanium

Single-chamber MEC reactors were built following Call and Logan (*30*). In this study, we increased the volumetric working capacity of the reactors to 100 mL (Fig. 1). Isomolded graphite plate (Graphitestore.com, Northbrook, IL, USA) was used as anode, connected to grade 2 Ti-wire (Ti-shop.com, London, UK). Stainless steel mesh, connected to stainless steel wire, served as a cathode. The electrodes were prepared following an existing protocol (*30*). External voltage was supplied at 0.7 V (P-3005 A; SUNSHINE Ltd., Guangzhou, PR China). Electrical measurements were continuously monitored over a 10 Ω resistor with a digital multimeter (APPA 109N; APPA, Taipei, Taiwan).

Anaerobiosis was achieved by vacuum-flush cycles of the reactor using ultra-high purity N_2_ gas, as demonstrated in the literature (*28*, *30*). Gases were filtered through 0.2-µm filters (hydrophobic politetrafluoroethylene sterile syringe filter; Axiva Sichem Biotech) during these cycles to maintain the sterility of the MECs. The setup was designed to be gas-tight and suitable for anaerobic culturing. To ensure proper sealing, we used a specific stopper designed for anaerobic culturing paired with a screw cap (GL-45 bromobutyl rubber stopper and GL-45 screw cap with aperture; DWK Life Sciences GmbH, Mainz, Germany). l-Cysteine HCl (Sigma-Aldrich, Merck) was added as a reducing agent in the medium to scavenge the leftover oxygen.

### Monitoring and data processing

Headspace gas was monitored on a gas chromatography-thermal conductivity detector (GC-TCD) unit (GC-8A; Shimadzu, Kyoto, Japan) on an activated carbon column with argon gas as the mobile phase. Injector and column temperatures were set to 130 and 100 ºC, respectively. Headspace gas was injected at a volume of 1 mL using a gas-tight syringe (Hamilton, Merck, Reno, NV, USA). Syringe volume was calibrated with pure H_2_ gas under normal atmospheric pressure (~101,325 kPa). Air, pure CH_4_ and pure H_2_ injections were made as peak identification controls.

Suspended bacterial growth was monitored periodically by measuring the absorbance at 600 nm on a spectrophotometer (UV-M90; BEL Engineering, Monza, Italy). At the end of the operation, we analysed the surface of the anodes by field emission scanning electron microscopy (FESEM; CMPFA Universitas Indonesia, Depok, Indonesia). Cells were fixed on the anodes following a modified chemical fixation protocol of Hrubanova *et al.* (*31*). Biofilms on the anodes were fixed using a 2.5% glutaraldehyde solution followed by repeated steps of ethanol dehydration, ending with air drying overnight at room temperature in a desiccator. Data presented in this study are average values of duplicate, independent MECs.

Soil microbiome was characterized at the end of the experiment through metagenomics approach on the culture medium using third-party services for gDNA extraction, next generation sequencing (NGS) of the V3-V4 region of the bacterial 16S rRNA, and bioinformatics analysis (Novogene, Singapore). The data is displayed as relative abundance. The phylogenetic tree of the designed consortium was generated from aligned sequences using MEGA7 software under the Clustal algorithm with a neighbour-joining method at a 1000 bootstrap value (*32*).

### Statistical analysis

Hydrogen data fit to model and calculations of p-value were carried out using GraphPad Prism v. 8.4.3 for macOS (*33*). Gompertz model used to describe H_2_ growth was obtained from the literature (*34*), formulated as follows:



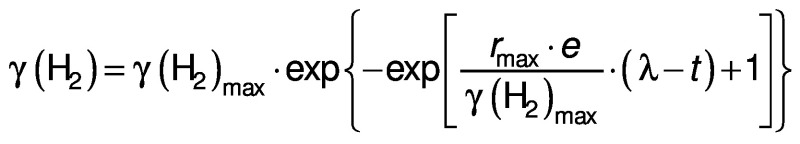



where *γ*(H_2_) stands for H_2_ concentration (mg/L), *γ*(H_2_)_max_ for maximum H_2_ concentration (mg/L), *r*_max_ for maximum H_2_ production rate (mg/(L·h)), *λ* for lag phase duration (h), and *t* for time (h).

## RESULTS AND DISCUSSION

### Biohydrogen production

**Fig. 2:**
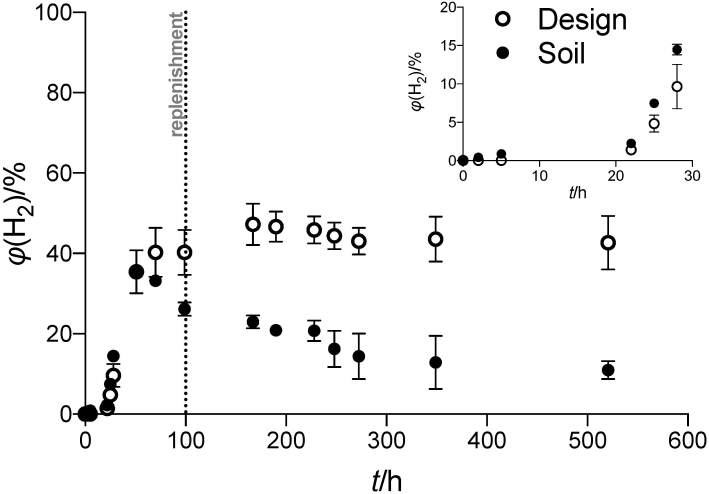
H_2_ composition in the headspace. The system was run without an external voltage supply in the first 22 h in the anode preparation stage. Medium replenishment at 10% working volume was carried out after the first current drop below 0.01 mA

Biohydrogen profile obtained using the system is shown in Fig. 2. At the beginning of the operation, the setup was run without external voltage. This period was designated as a preparatory period for the inoculum, during which no H_2_ was produced (0–22 h) due to the lack of energy available to overcome the thermodynamic barrier of H_2_ generation in MECs. Once the system was run with external voltage, both setups produced H_2_ exponentially_._ This is clear if we examine the 0–30 h period. MECs inoculated with native soil bacteria exhibited a decline after peaking at 35% of headspace H_2_ concentration at 51 h of operation. The decrease in the headspace concentration when using soil inoculum started prior to medium replenishment. It continued even after the medium was replenished, unlike with designed consortium, which responded to medium replenishment with a slight increase in H_2_ concentration. On the other hand, headspace H_ 2_ concentration with the designed microbial consortium stabilized at ~43% at the end of the observation (270–520 h), slightly under its peak value of 47% at 167 h. The two microbial consortia started to differ significantly in H_2_ concentration at 99 h of the operation (p<0.05) and continued to do so until the end despite exhibiting similar profiles in the period leading up to this point (0–60 h).

The decrease of H_2_ concentration in the headspace is likely to be attributed to a transformation into methane (methanogenesis) that is common in this type of reactor configuration. Methanogenesis was often found as a cause of failure to obtain biohydrogen in MECs. In the original study that inspired our setup, total conversion of H_2_ to CH_4_ occurred, leading to undetected quantities of H_2_ in the headspace at the end of the culture (*30*). Our work has managed to maintain H_2_ at a higher level throughout, ~45% with the designed consortium and ~18% with the native soil consortium over the observed culture period. Other works tried to avoid methanogenesis by physical or chemical means like adding antibiotics/inhibitors (*35*, *36*), intermittent oxygenation (*37*) or ultraviolet irradiation (*38*). Each of these methods has its own benefits and limitations. For example, the addition of antibiotics in the culture medium poses a risk of a potential spill of the resistance trait over to the environment if care is lacking.

**Fig. 3:**
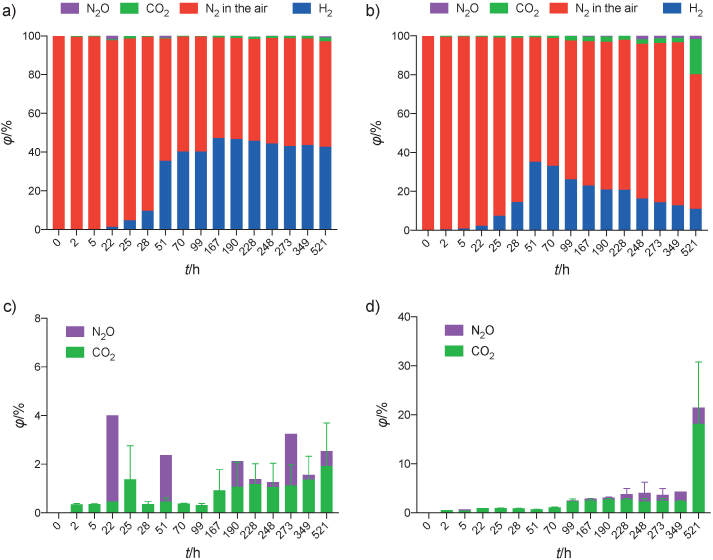
Headspace gas profile from the two microbiomes: a) all gases with designed consortium and b) all gases with soil consortium. Trace amounts of gases from: c) designed and d) soil consortia. Data are expressed as relative abundance over total detected gas concentration

Aside from H_2_, several other components of headspace gas were also detected (Fig. 3). These components are H_2_, N_2_ in the air, CO_2_ and N_2_O. Our method has a limitation in separating air into its molecular components of N_2_ and O_2_. Hence N_2_ is referred to in the results as ‘N_2_ in the air’. Additionally, O_2_ is practically absent from the system due to the vacuum-flush cycle and the addition of oxygen-scavenging species in the media.

N_2_O is an intermediary metabolite in the denitrification process (*39*). The existence of this gas in the headspace suggests that nitrate-reducing activity was present in the system. The presence of N_2_ in the system is expected since the gas is used in the beginning to purge O_2_ out of the system. Hence, it cannot be used as a marker for a complete denitrification process despite being the end product of the pathway.

Methane was interestingly absent from detection in this study. A possible explanation could be the transformation of methane into other metabolites like CO_2_ in anaerobic methane oxidation, which could be the mechanism behind the significant jump in CO_2_ concentration at the end of the cycle with soil MEC. The presence of CO_2_ in MECs is otherwise normal since the breakdown of organic matter/substrate in the anode often results in CO_2_ release (*4*). Methanogenic bacteria and denitrifying bacteria can interact in multiple metabolic pathways in the environment. For example, denitrifying anaerobic methane oxidation (DAMO) can be found in nature (*40*), which presents an opportunity for the same process to occur in the setup, leading to the transformation of produced methane to carbon dioxide. DAMO is currently of interest as a competing pathway to reduce methane in MECs (*24*). In the future, further characterization of the metabolic processes in the system is needed to confirm the presence of this interaction.

**Fig. 4:**
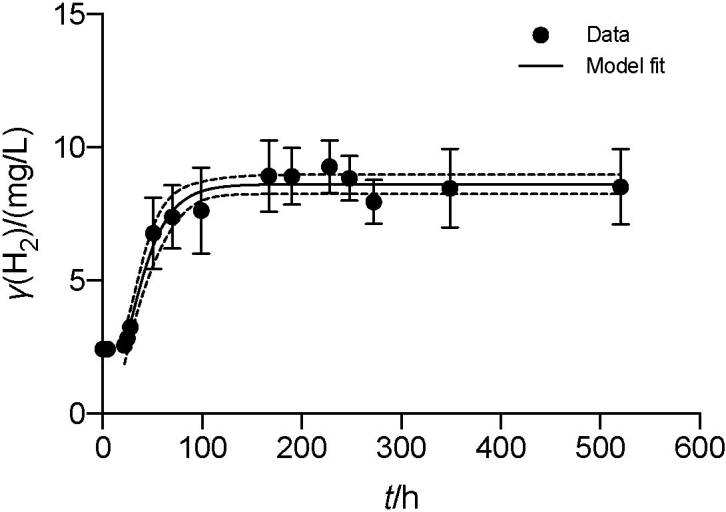
Hydrogen profile model fit for the designed consortium based on Gompertz growth model; data presented after external voltage were supplied to the system. Dotted lines represent the model's confidence band (CI=95%)

To validate the H_2_ profile obtained in this study for the designed consortium, which resembles a regular growth curve of batch culture, we chose a common growth model to fit the data. Natural soil consortium possesses a different H_2_ profile, likely due to the consumption of H_2_, which does not suit the chosen growth model well. In batch culture, bacterial growth rate followed these well-known stages: lag, acceleration, exponential, slowing down, stationary and death phases. Growth models may take a linear form like Monod or non-linear forms, such as Gompertz and logistic models (*41*). The Gompertz model was selected for this study, relying on simple information related to H_2_ evolution in the system. Meanwhile, Monod was unsuitable since it requires additional information related to substrate consumption. Nonetheless, the H_2_ production in MEC with designed consortium can be described well using Gompertz model as presented in Fig. 4. The modified Gompertz model was first formulated by Zwietering *et al.* (*42*) and adjusted to describe H_2_ in newer studies (*34*).

To consider the period of preparation before running the MEC, this period was excluded from the model (0–22 h). The correlation coefficient (R^2^) of the model fit was 0.973. Using the model, several parameters were obtained: *r*_max_ (mg/(L·h)), *γ*_max_ (mg/L) and *λ* (h). To determine the rate of H_2_ generation *r*_max_ can be used. *γ*_max_ corresponds to the maximum H_2_ concentration, while *λ* is related to the lag phase after initialization of the system. For this study, model fit values for *r*_max_, *γ*_max_, and *λ* were 0.247 mg/(L·h), 8.605 mg/L and 20.98 h, respectively. A confidence band based on a confidence interval of 95% was used to graphically present the true location of the curve (Fig. 4). The fit of the model, assessed from its correlation coefficient, corresponds well to more than 20 values presented by Wang and Wan (*34*) in the range of 0.90–1.0 despite coming from a different setup than the batch fermentation method that is traditionally used to produce biohydrogen. This result suggests that H_2_ production in MEC can be modelled like a traditional batch fermentation process, given that the H_2_ profile matches those of ordinary batch growth/processes.

### Microbiome characterization

**Fig. 5:**
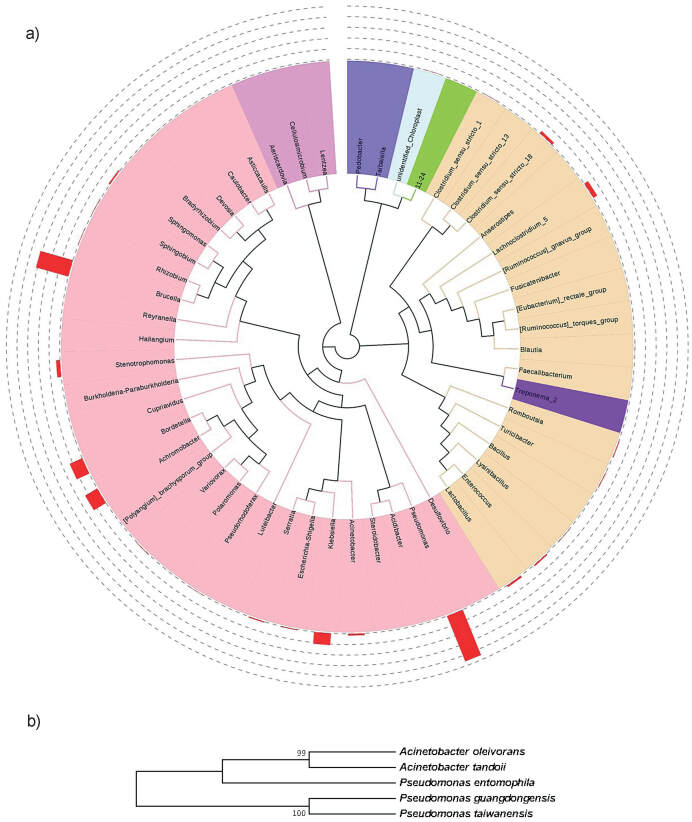
Comparison of microorganisms in the microbiomes in: a) soil consortium, along with relative abundance up to genus level, and b) composition of the designed consortium, presented in a phylogenetic tree (constructed from aligned sequences using neighbor-joining method and 1000 bootstrap value)

Characterization of the soil community is available in Fig. 5. Soil bacteria were detected based on metagenomics approach. This approach was chosen since it can provide a more expansive overview of genetic materials present in environmental samples, including from microbes that may be difficult to isolate and preserve in laboratory settings. The sensitivity of the method provides insight into the complexity of soil microorganisms.

The MEC inoculated with soil contained mostly bacteria of the class Gammaproteobacteria (48.54%) that includes both *Pseudomonas* and *Acinetobacter* genera. *Pseudomonas* and *Acinetobacter* are classified as Gram-negative bacteria, much like the other genera dominating natural soil consortia. The medium used in this study contains high nitrate concentration, requiring bacteria to possess the necessary adaptive ability to survive. For designed consortium, the bacteria were preselected based on their capability to survive in a nitrate-rich environment as well as to metabolize nitrate using the denitrification pathway (*25*). The use of denitrifying bacteria as competitors to methanogenesis is based on the idea that metabolites released from denitrification may act as inhibitors to methanogens in soil samples (*17*).

Differences in H_2_ content in the headspace can be attributed to the microbiome inside the system (Fig. 2). Soil microbiome was dominated by several genera, in descending order: *Pseudomonas*, *Brucella*, *Achromobacter*, *Bordetella*, *Klebsiella*, *Lachnoclostridium* 5, *Stenotrophomonas*, *Clostridium sensu stricto* 18, *Lactobacillus* and *Acinetobacter*. On the other hand, the designed consortium consists only of several species belonging to two genera, *Pseudomonas* and *Acinetobacter*, which are also present among the top ten genera in the soil microbiome. This is in agreement with the fact that the two genera were originally isolated from soil samples in Indonesia. Hence, we drastically reduced the complexity of the community by reducing a rich source of microbiome to only two genera. Future adjustments of the composition of the designed consortium could include well-documented electroactive bacteria like *Geobacter* to further facilitate electron transfer and H_2_ production (*43*, *44*).

### Electrode characterization

**Fig. 6:**
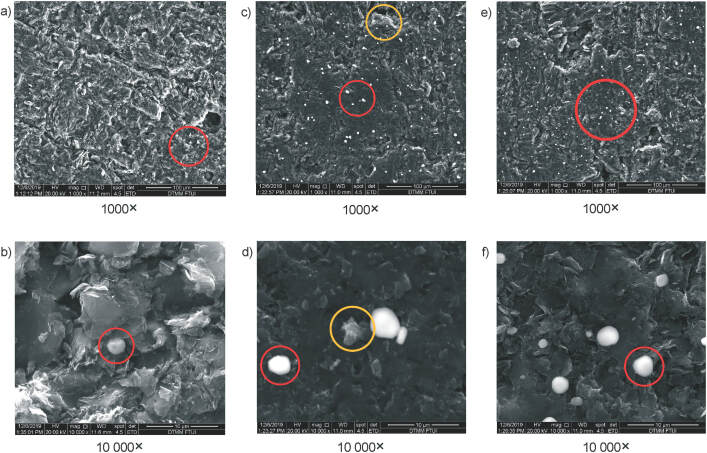
Anode surface characterization using field emission scanning electron microscopy (FESEM) in: a and b) plain anode material, c and d) soil microbiome, and e and f) designed consortium. Red circle=intact structure; yellow circle=degraded structure

Exoelectrogenic microorganisms may transfer electrons to their environment by physical or chemical means. Physical mechanisms include the presence of structural nanowires, a term for electrically conductive pili (*45*). Chemically, electron transfer may occur through mediators secreted by the bacteria, *i.e.* pyocyanin by *Pseudomonas aeruginosa* (*46*). In MECs, electroactive species present in the medium can spontaneously attach themselves to form biofilms on the surface of the anodes (*47*). In this study, the physical states of anodes post-operation were analysed by SEM imaging. The anodes were treated prior to imaging following a modified approach from existing literature (*31*) to prevent structural degradation of biofilms, as presented in Fig. 6. Chemical methods utilized to fix the biomass prior to imaging involve repeated washing, which may degrade the extracellular matrix of biofilms present on the surface (circled in yellow). However, it is a more straightforward method than cryotreatment, and suitable for surface imaging (*31*).

The presence of biofilms on the anodes suggests that in both consortia physical transfer of electrons is possible. Additionally, given that *Pseudomonas* comprise the majority of the designed consortium and a major fraction of the soil consortium, it is also possible that pyocyanin-secreting species are present, hence allowing mediator-based electron transfer. In future studies, it would be interesting to analyse and compare the composition of microbiomes found on the surface of the anodes with microbes suspended freely in the medium. A similar imaging approach was used in literature (*23*) where they managed to show nanowires used for direct interspecies electron transfer. This approach is interesting to use for consortium with distinct morphological differences. In our case, the bacteria were morphologically similar.

## CONCLUSIONS

Designed consortium, preselected for its ability to grow in a nitrate-rich environment and carry out the denitrification process, performed better than native soil consortium for biohydrogen production. H_2_ profile was sustained for a longer period without signs of transformation to methane, a familiar yet undesired phenomenon in single-chamber microbial electrolysis cells (MECs). The single-chamber configuration of MECs presents advantages over multi-chamber configurations thanks to its simplicity. Here we exploit this configuration to produce biohydrogen in a simple laboratory-scale setup. The results presented in the study suggest that reducing microbiome complexities in the inoculum may be beneficial to avoid undesired transformative pathways in MECs. This effect is evident when using preselected or ‘designed’ communities for specific characteristics. In this study, we demonstrate the avoidance of methanogenesis by co-culturing denitrifying bacteria in MECs with prior understanding of the inhibitory effect of denitrification on methanogenic bacteria. Further studies are needed to better understand the biological aspect of this phenomenon by utilizing more powerful analytical tools to explore the complexity of the two consortia better. It would also be interesting to optimize the designed microbiome's performance for biohydrogen generation using other consortium formulations and different growth media, *i.e.* wastewater as nitrate-rich growth medium.
